# A Group of *ent*-Kaurane Diterpenoids Inhibit Hedgehog Signaling and Induce Cilia Elongation

**DOI:** 10.1371/journal.pone.0139830

**Published:** 2015-10-06

**Authors:** Shiyou Jiang, Jiacheng Du, Qinghua Kong, Chaocui Li, Yan Li, Handong Sun, Jianxin Pu, Bingyu Mao

**Affiliations:** 1 State Key Laboratory of Genetic Resources and Evolution, Kunming Institute of Zoology, Chinese Academy of Sciences, Kunming, China; 2 State Key Laboratory of Phytochemistry and Plant Resources in West China, Kunming Institute of Botany, Chinese Academy of Sciences, Kunming, China; 3 Kunming College of Life Science, University of Chinese Academy of Sciences, Kunming, China; Indiana University School of Medicine, UNITED STATES

## Abstract

The Hedgehog (Hh) signaling pathway plays important roles in the tumorigenesis of multiple cancers and is a key target for drug discovery. In a screen of natural products extracted from Chinese herbs, we identified eight *ent*-Kaurane diterpenoids and two triterpene dilactones as novel Hh pathway antagonists. Epistatic analyses suggest that these compounds likely act at the level or downstream of Smoothened (Smo) and upstream of Suppressor of Fused (Sufu). The *ent*-Kauranoid-treated cells showed elongated cilia, suppressed Smo trafficking to cilia, and mitotic defects, while the triterpene dilactones had no effect on the cilia and ciliary Smo. These *ent*-Kaurane diterpenoids provide new prototypes of Hh inhibitors, and are valuable probes for deciphering the mechanisms of Smo ciliary transport and ciliogenesis.

## Introduction

The Hedgehog (Hh) pathway plays fundamental roles in embryonic development, and aberrantly activated Hh signaling drives the formation of multiple cancers, particularly basal cell carcinoma (BCC) and medulloblastoma (MB) [1]. In the past decade, major progress has been made in Hh pathway targeted cancer therapies with Hh pathway antagonists (HPAs). Several Smoothened (Smo) antagonists are in clinical trials for treating a variety of cancers. For example, vismodegib (GDC–0449, Roche) is the first HPA approved by the FDA for treating BCC [2]. However, acquired resistance to vismodegib was soon observed in MB and BCC patients, resulting in cancer relapse [3, 4]. Genomic analysis revealed that the predominant mechanism of resistance was Smo mutations that disrupted vismodegib binding with the pocket of Smo [5, 6]. Thus, it has been widely suggested to develop new HPAs that target different molecules.

In mammals, Hh signal transduction depends strictly on the primary cilium. Many of the crucial components in the mammalian Hh pathway are concentrated within cilia, and most of the pivotal signaling transduction events take place in the cilia. Thus, the normal structures and functions of cilia are essential for Hh signal transduction [1, 7, 8]. In the absence of Hh ligands, the Hh receptor Patched (Ptch) locates to the cilia and prevents ciliary accumulation of Smo to repress the pathway. When Hh binds Ptch, Ptch is internalized and removed from the cilia, allowing Smo to enter and accumulate in the cilia, where it activates the transcriptional effector Gli by relieving its inhibition by Suppressor of Fused (Sufu) [1, 7]. The primary cilia can either promote or inhibit BCC and MB development depending on the oncogenic events that have occurred, indicating that cilia could be a potential diagnostic marker and therapeutic target in Hh-related cancers [9, 10].

Currently, most HPAs are synthetic compounds with similar structural skeletons and limited diversity [11]. Plant natural products, which possess enormous structural and chemical diversity, have been a continuing source for drug discovery, often showing surprising bioactivities [12]. The first Hh pathway inhibitor, Cyclopamine (Cyc), is a natural product from corn lilies that binds to the 7-transmembrane domain of Smo [13, 14]. To discover new HPAs, we conducted a screen of natural products from herbs and identified a group of *ent*-Kaurane diterpenoids that inhibit the Hh pathway and promote cilia elongation.

## Materials and Methods

### Cell culture

NIH 3T3 (ATCC, CRL-1658^TM^), Shh light II (ATCC, CRL-2795^TM^) [14], HEK 293 (ATCC, CRL-1573^TM^), HEK 293W [15] and Sufu^-/-^ MEFs [16] cells were grown in DMEM supplemented with 10% (v/v) fetal bovine serum (FBS), penicillin, and streptomycin. Fibroblast-derived cells were prevented from becoming too confluence before propagation to maintain the property of contact inhibition. Shh light II cells were derived from NIH 3T3 cells stably transfected with the following dual luciferase reporters: (1) Gli-responsive firefly luciferase and (2) thymidine kinase-derived *Renilla* luciferase [14]. HEK 293W cells were derived from HEK 293 cells stably transfected with Wnt3a and the following dual luciferase reporters: (1) Wnt responsive SuperTOPflash firefly luciferase and (2) simian virus 40-*Renilla* luciferase [15]. The Sufu^-/-^ MEFs were originally derived from Dr. Rune Toftgård’s lab [16] and were kindly provided by Dr. Steven Y. Cheng [17] with the permission of Dr. Rune Toftgård.

### Reagents

SAG, cytochalasin D, 5Z-7-Oxoeaneol, GANT58, EGCG and MG132 were purchased from Sigma, vismodegib from Selleck Chemicals, 20(S)-hydroxycholesterol (20(S)-OHC) from Cayman Chemicals, Bodipy-Cyclopamine from Toronto Research Chemicals, and vinblastine and AZ-TAK1 from Santa Cruz Biotech.

### Isolation of Natural Products

Kamebakaurin, kamebakaurinin [18], phyllostachysin H [19], calcicolin A [20], tenuifolin A, tenuifolin I [21], adenanthin C and adenanthin G [22] were isolated from the genus *Isodon* as described. Longipedlactone L [23] was isolated from *Kadsura ananosma* and longipedlactone H [24] from *Kadsura longipedunculata* as described. The plants were collected in Yunnan, China; no permission was required.

### Hh Pathway Dual Reporter Assay

Shh light II cells were propagated on white 96-well assay plates (Corning) and grown to extreme confluence. Then the medium was changed to 0.5% FBS/DMEM medium with various compounds for 30 hr. The cells were lysed and the firefly and *Renilla* luciferase activities were measured using the Bright-Glo reagents (Promega) on a Fluoroskan Ascent (Thermo Fisher). All of the samples were conducted in triplicate. The Gli-firefly/*Renilla* luciferase ratio represented the Hh pathway activity. For Wnt reporter analysis, HEK 293W cells were seeded on 96-well plates at 60% confluence, treated with the compounds for 1 day, and then lysed to measure the reporter activities.

### Bodipy-Cyclopamine Competition Binding Assay

Bodipy-Cyclopamine competition binding assays were conducted as previously described [13] with modifications. HEK 293 cells were seeded onto 24-well plates and transfected with Smo-mCherry using Fugene HD (Roche) at 70% confluence. After 2 days of Smo expression, the cells were incubated with 10 nM Bodipy-Cyc and 10 or 20 μM of various HPAs for 1 hour (100 nM of SAG was used as a positive control). Then, the cells were fixed and stained with Hoechst 33342 to visualize the nuclei. All of the images were captured under the same exposure conditions using a 10x objective with an Olympus FV1000 confocal microscope. At least five images were taken from each sample with similar Smo-mCherry expression levels. Fluorescence intensities of each image were quantitatively measured using ImageJ (NIH).

### Real-time PCR Analysis of Hh Target Gene Expression

NIH 3T3 cells and Sufu^-/-^ MEFs were grown to confluency and their medium were changed to 0.5% FBS medium diluted with various HPAs for 30 hr. Total RNA was extracted and purified using TRIzol (Invitrogen) according to the standard protocol. Next, 1 μg of total RNA from each sample was reversely transcribed to cDNA with random hexamer primer (Fermentas). The mRNA levels of mouse *Gli1*, *Ptch1*and *Gapdh* were quantified using LightCycler 480 SYBR Green I Master (Roche) on a LightCycler 480 system (Roche). The following primers were used: (1) Gli1, 5’- CCAAGCCAACTTTATGTCAGGG–3’ and 5’- AGCCCGCTTCTTTGTTAATTTGA–3’; (2) Ptch1, 5’- CGAGACAAGCCCATCGACATTA–3’ and 5’- AGGGTCGTTGCTGACCCAAG–3’; and (3) Gapdh, 5’- TGTGTCCGTCGTGGATCTGA–3’ and 5’- TTGCTGTTGAAGTCGCAGGAG–3’.

### Cilia and Ciliary Smo Analysis

For *de novo* ciliogenesis and ciliary Smo analysis, NIH 3T3 cells were grown to confluence, and then their medium was changed to serum starvation medium (0.5% FBS/DMEM) with various compounds for 30 hr. To analyze the effects of *ent*-Kauranoids on mature cilia, the confluent cells were first incubated in serum starvation medium for 1 day to fully induce cilia formation before the compounds were added for an additional 30 hr. Cilia and Smo immunostaining was performed according to previously reported procedures [25]. The following antibodies were used for the immunostaining: rabbit polyclonal anti-Arl13b (17711-1-AP, Proteintech), mouse monoclonal anti-Smo (sc–166685, Santa Cruz), mouse monoclonal anti-acetylated α-tubulin (T7451, Sigma), and donkey AlexaFluor 488 or 594 conjugated secondary antibodies (Invitrogen).

Images were acquired with identical setting parameters, using 6-plane Z-stacks (1 μm step size) to encompass all of the cilia and Smo in the field with a 100X oil immersion objective on an Olympus FV1000 laser confocal microscope. Images of the Z-stacks were summed as final micrographs for quantitative assessment with the method of maximal intensity projections using Image-Pro Plus 6 (Media Cybernetics). To measure the length of the cilia, images were calibrated with scale ruler, the cilia contour were outlined using the segmented line tool, and the lengths of cilia were measured with ImageJ. To quantitatively analyze the ciliary Smo, the cilia were manually outlined with a mask in the Arl13b channel and the Smo fluorescence intensities were then measured in the Smo image channel. The total signal for a single cilium was taken as one data point. Approximately 100 cilia and ciliary Smo were measured from ten images for each group.

### Statistical Analysis

Calculation of the mean, SD and IC_50_ with the Student’s *t* test and one-way ANOVA analysis were performed with GraphPad Prism 6.

### Mitotic Spindle Assembly Assay

Mitotic spindle assembly assay was performed as previously reported [25]. Briefly, NIH 3T3 cells were enriched at metaphase with 15 μM MG132 for 90 min, followed by a 60 min incubation with 15 μM MG132, and 50 or 25 μM kamebakaurin. Cells were fixed by methanol for 10 min at -20°C, and immunostained with mouse monoclonal anti-α-tubulin (T9026, Sigma) and rabbit polyclonal anti-pericentrin (ab4448, Abcam) antibodies. Approximately 100 metaphase spindles were scored.

### Tubulin and Actin Polymerization Assays

The effect of kamebakaurin on tubulin and actin polymerization was performed using a Tubulin Polymerization Assay Kit (BK011P, Cytoskeleton) and an Actin Polymerization Biochem Kit (BK003, Cytoskeleton), respectively, with the manufacturer’s guidance.

## Results

### A group of *ent*-Kaurane Diterpenoids and Triterpene Dilactones Inhibit Hh signaling

**Fig 1:**
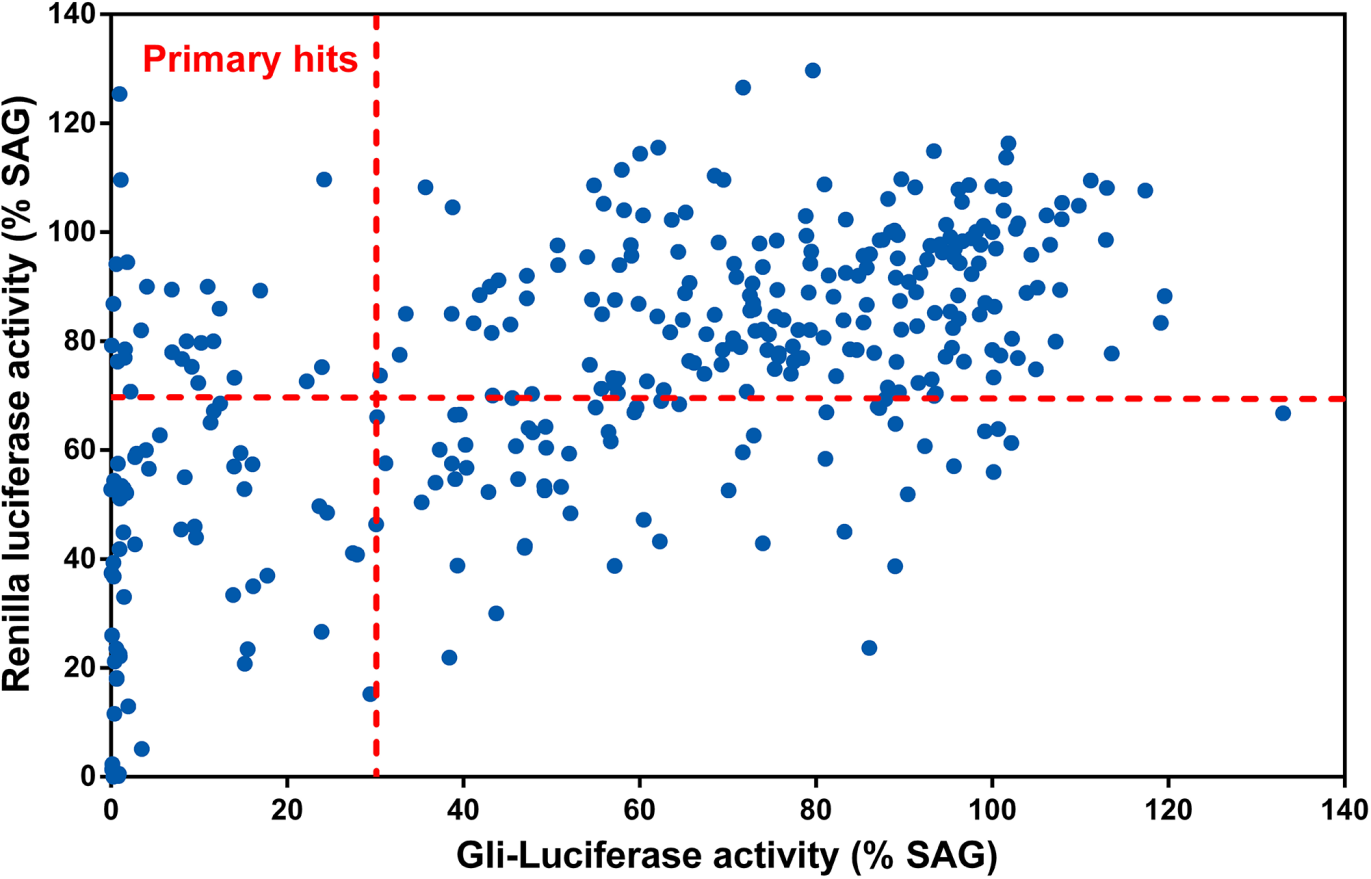
The activity distribution of the herbal natural products in the screen for Hh pathway antagonists. The primary screen for Hh pathway antagonists was performed in Shh light II cells treated with 10 μM natural products (stock concentration in DMSO at 10 mM) with 150 nM SAG for 30 hr. The selected primary hits displayed an inhibitory effect on the Gli-reporter of more than 70% and *Renilla* activity of less than 30%. Activities of the compounds were normalized to SAG.

**Fig 2:**
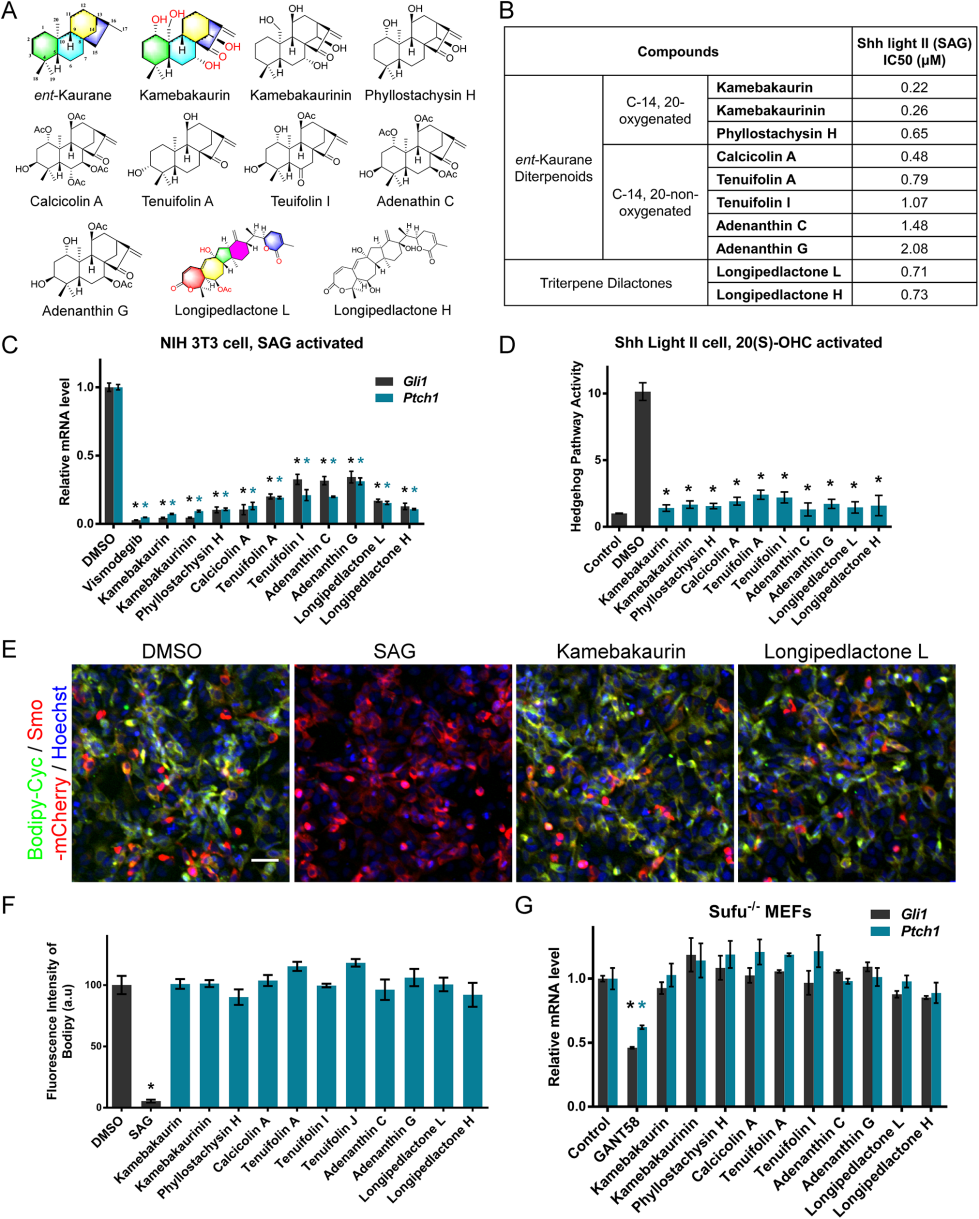
Identification of a group of *ent*-Kaurane diterpenoids and triterpene dilactones as Hh pathway antagonists. **(A)** Chemical structures of the *ent*-Kaurane diterpenoids and the triterpene dilactones. **(B)** Half maximal inhibitory concentrations (IC_50_) of the HPAs in the Hh reporter assays activated by SAG. **(C)** The HPAs inhibit SAG activated expressions of the Hh target genes *Ptch1* and *Gli1*. **(D)** The HPAs suppress 20(S)-OHC-activated Hh signaling. **(E)** The HPAs do not compete with Bodipy-Cyc for Smo binding. Scale bar: 50 μm. **(F)** Quantitation of the intensities of bound Bodipy-Cyc to Smo-expressing cells treated with the HPAs. **(G)** Unlike GANT58, which targets Gli1 downstream of Sufu, the HPAs do not suppress the activation of the Hh pathway in Sufu^-/-^ MEFs. The relative expression levels of the Hh target genes *Ptch1* and *Gli1* were determined. The data in C, D, F and G are expressed as the mean ± SD, and the HPAs were used at 10 μM. Asterisks indicate *p* < 0.05 for individual compounds vs. DMSO or control.

To search for novel HPAs, we conducted a screen of 500 natural products extracted from medicinal plants with a cell-based reporter assay using the Smo agonist SAG [26] to activate Hh signaling (Fig 1). During this screen, a group of *ent*-Kaurane diterpenoids and triterpene dilactones were identified. The 8 *ent*-Kaurene diterpenoids, kamebakaurin, kamebakaurinin [18], phyllostachysin H [19], calcicolin A [20], tenuifolin A, tenuifolin I [21], adenanthin C and adenanthin G [21], were isolated from plants of the genus *Isodon*. The 2 triterpene dilactones, longipedlactone L [23] and longipedlactone H [24], were isolated from *Kadsura ananosma* and *Kadsura longipedunculata*, respectively (Fig 2A). Plants from the genus *Isodon* are rich with diverse highly oxygenated *ent*-Kauranoids that have been used as anti-tumor, antibacterial and anti-inflammatory agents in Chinese folk medicine [27]. Triterpenoids from plants from the genus *Kadsura* have been reported to have anti-tumor and anti-HIV activities [21]. The chemical structures of the ten HPAs (Fig 2A) are different from all known Hh antagonists and represent novel prototypes of Hh inhibitors.

The 8 *ent*-Kauranoids share an *ent*-Kaurane skeleton with different hydroxyl and acetoxyl substituents (Fig 2A) and are categorized into C–14, 20-oxygenated and non-oxygenated groups, with the oxygenated compounds having stronger activities (Fig 2B, S1 Fig). These results reflect the structure-activity relationships of *ent*-Kauranoids that inhibit the Hh pathway. The 2 triterpene dilactones, longipedlactones L and H, had similar structures and activities (Fig 2A and 2B, S1 Fig).

Gli1 and Ptch1 are key components of the Hh pathway and are also Hh pathway target genes [1]. The HPAs significantly inhibited *Gli1* and *Ptch1* expression stimulated by SAG (Fig 2C). In contrast, these HPAs had no clear inhibitory effect on Wnt signaling (S2 Fig). The HPAs also repressed Hh signaling induced by the Smo agonist 20(S)-OHC, which is different from SAG and binds to the cysteine-rich domain of Smo [28] (Fig 2D). The evidence suggests that HPAs act at the level or downstream of Smo. Cyclopamine inhibits Hh signaling by binding to the heptahelical bundle of Smo, which can be competed away by most known Smo-targeting HPAs [11, 13]. In a competition experiment, the *ent*-Kauranoids and triterpene dilactones showed no effect on the binding of Bodipy-labeled Cyc to Smo-expressing cells (Fig 2E and 2F), suggesting that they do not bind the Cyc-binding pocket of Smo. Sufu is a negative regulator of the Hh pathway that is downstream of Smo and Sufu knockout results in activated Hh signaling [16, 29]. Real-time PCR results showed that the *ent*-Kauranoids and dilactones had no clear inhibitory effect on *Gli1* and *Ptch1* expression in Sufu^-/-^ MEFs (Fig 2G), suggesting that they likely work upstream of Sufu. Given that kamebakaurin has been reported to inhibit the nuclear factor *κ*B (NF-*κ*B) pathway [30], we tested whether established NF-*κ*B inhibitors, AZ-TAK1 [31] and 5Z-7-Oxoeaneol [32], could repress Hh signaling. As they both showed no clear effect on the expression of the Hh reporter stimulated by SAG (S3 Fig), we hypothesize that *ent*-Kauranoid HPAs do not indirectly inhibit Hh by repressing the NF-κB pathway.

### *ent*-Kauranoids Elongate Primary Cilia and Inhibit Ciliary Accumulation of Smo

**Fig 3:**
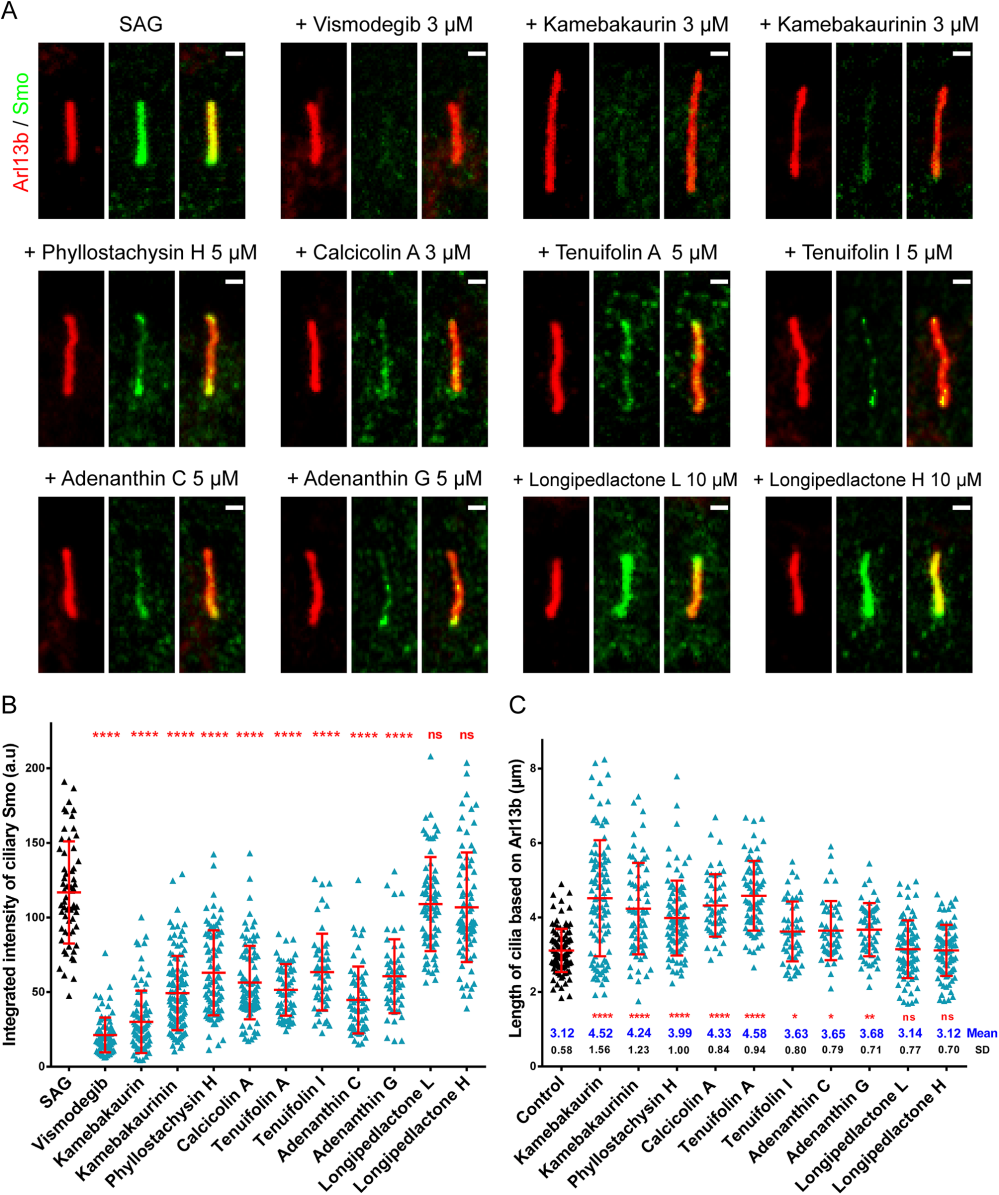
The *ent*-Kauranoids elongate cilia and inhibit ciliary accumulation of Smo. **(A)** Representative images of cilia (stained with the ciliary protein Arl13b, red) and ciliary Smo (green) in NIH 3T3 cells treated with either SAG alone or together with the HPAs. The compounds were used at the concentration with the maximum effect on cilia elongation while showing no clear toxic effects on the cells. Scale bar: 1 μm. **(B)** Quantitative assessment of the integrated intensity of ciliary Smo from the images. **(C)** Quantitative analysis the length of cilia from the Arl13b images. Asterisks indicate significance using one-way ANOVA analysis (*, *p* < 0.05; **, *p* < 0.01; ****, *p* < 0.0001; ns, not significant). The data are represented as the mean ± SD.

Smo enrichment in the cilia is critical for the activation of Hh pathway [7, 8]. Thus, we evaluated the effects of the HPAs on the ciliary accumulation of Smo induced by SAG. The *ent*-Kauranoids, but not the triterpene dilactones, remarkably reduced the levels of ciliary Smo (Fig 3A and 3B). Unexpectedly, the cilia were dramatically elongated in cells treated with the *ent*-Kauranoids but not the dilactones (Fig 3A and 3C). The intraflagellar transport (IFT) complex proteins affect the Hh pathway downstream of Smo and upstream of Gli, which are essential for the assembly and maintenance of primary cilia and ciliary transportation [7, 33]. We speculate that the *ent*-Kauranoids likely act at similar levels as IFT, by affecting ciliogenesis and ciliary transport of cargo.

**Fig 4:**
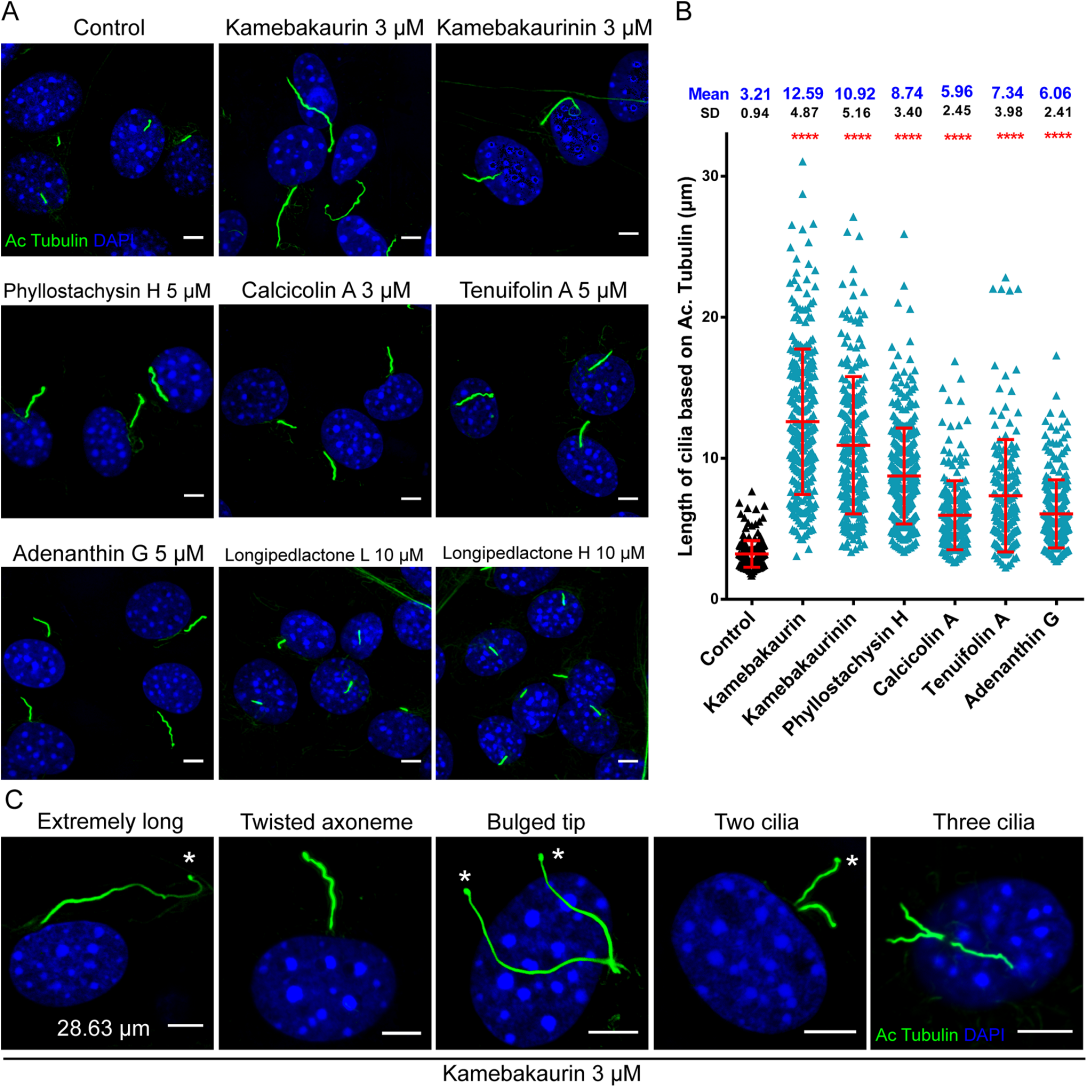
The *ent*-Kauranoids remarkably elongate mature cilia. **(A)** Representative images of the effects of HPAs on well-formed mature cilia. The cilia were visualized by staining acetylated α-tubulin (Ac Tubulin). **(B)** Quantitative analysis the length of cilia from the Ac Tubulin images. Asterisks indicate significance with one-way ANOVA analysis (****, *p* < 0.0001). The data are presented as the mean ± SD. **(C)** Representative images of observed cilia malformations. Asterisks indicate bulged tips. Scale bar: 5 μm.

In the above experiments, the *ent*-Kauranoids were directly added after the cells reached confluence, when *de novo* ciliogenesis was to start. We further investigated their effects on well-formed mature cilia. The confluent cells were starved for one day to fully induce cilia formation before the drugs were added. Under such conditions, the *ent*-Kauranoids showed more prominent and variable effects on the elongation of the cilia as are shown by quantitative analyses of staining for the ciliary axoneme marker acetylated α-tubulin (Fig 4A). For example, cilia in kamebakaurin- and kamebakaurinin-treated cells were 12.59 ± 4.87 μm and 10.92 ± 5.16 μm in length, which were 3.92- and 3.40-fold of normal cilia, respectively. Cells treated with other *ent*-Kauranoids also had elongated cilia, ranging from 6.06 to 8.74 μm, while the cilia in cells treated with triterpene dilactones were normal (Fig 4A and 4B). Moreover, cilia with abnormal morphology, such as cilia with twisted and wave-like axoneme or bulged tips or cells with 2 or 3 cilia, were frequently observed (Fig 4C). KIF7 is a cilia-associated protein belonging to the kinesin family that plays a role in the hedgehog signaling pathway [34–37]. Cells with mutated Kif7 grow long and twisted cilia, similar to the cells treated with *ent*-Kauranoids. However, Kif7 mainly regulates the Sufu-Gli complex without affecting Smo [34–37], making it unlikely to be the target of the *ent*-Kauranoids.

### Kamebakaurin Disturbs Spindle Assembly and Chromosome Congression

**Fig 5:**
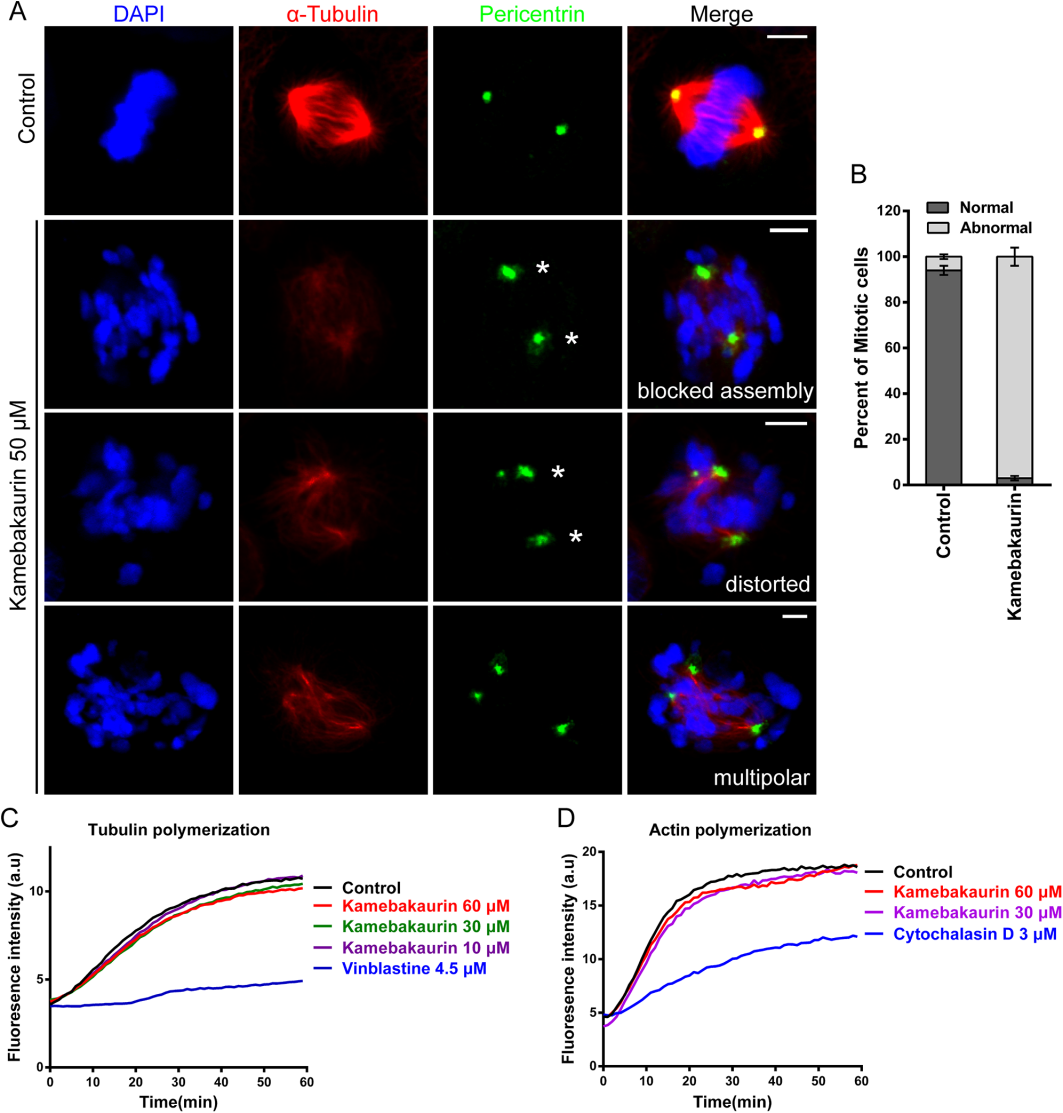
Kamebakaurin causes multiple spindle defects and misaligned chromosomes. **(A)** Metaphase spindle defects in NIH 3T3 cells treated with kamebakaurin (50μM). Asterisks indicate diffused pericentrin distribution. Scale bar: 5 μm. **(B)** Quantification of the percentage of cells with abnormal spindles in kamebakaurin- (50μM) or DMSO (control)-treated groups. More than 100 cells were counted in each group. The data are presented as the mean ± SD. **(C, D)** Kamebakaurin has no effect on the polymerization of tubulin or actin in *in vitro* assays. Vinblastine and cytochalasin D are known tubulin and actin inhibitors, respectively.

Kamebakaurin is the most potent *ent*-Kauranoid that we identified in Hh inhibition and cilia elongation experiments. Interestingly, when treated with kamebakaurin, cells with binuclei or micronuclei were frequently observed to undergo abnormal mitosis (S4 Fig). Thus, we examined the mitotic spindle structures in kamebakaurin-treated cells, which were enriched to metaphase by MG132 [25]. Indeed, kamebakaurin treatment led to defective (distorted, multipolar) spindles with poor spindle microtubule assemblies and spindles lacking of the fusiform shape (Fig 5A). The congression of chromosomes often failed when the chromosomes misaligned with no attachment to the microtubules. In control groups, cells incubated with vehicle alone had normal spindle morphology and chromosomes congression (Fig 5A and 5B). The distribution of the pericentriolar matrix protein pericentrin became more diffused (Fig 5A, S5 Fig). At a lower concentration (25 μM), kamebakaurin induced clear but less severe spindle malformation in treated cells. (Fig 5A, S5 Fig). We next examined whether kamebakaurin inhibits spindle formation by interfering with the polymerization of microtubules and actin. Kamebakaurin showed no effects on either microtubule or actin dynamics *in vitro* (Fig 5C and 5D), suggesting that it likely targets protein(s) other than tubulin and actin.

## Discussion

In the last decades, major progress has been made in the development of small molecules that specifically inhibit the Hh signaling pathway. Currently, most identified HPAs target Smo, several of which are being tested in clinical trials for the treatment of multiple types of cancer [2]. Unfortunately, acquired resistance to Smo inhibitor has already been reported in patients with advanced BCC and MB [3, 4]. Thus, Hh pathway inhibitors acting downstream of Smo could be more promising for such patients. In our study, we identified two triterpene dilactones and a group of *ent*-Kauranoids as potent Hh pathway inhibitors with distinct mechanisms of action. To our knowledge, this is the first report of HPAs with triterpene and *ent*-Kauranoid chemical backbones.

Further analyses suggest that the *ent*-Kauranoids likely act at the level or downstream of Smo, and upstream of Sufu. Interestingly, the *ent*-Kauranoids can inhibit cilia trafficking of Smo and promote cilia elongation, whereas the triterpene dilactones have no such effects. Kamebakaurin, the most potent compound identified in our screen, also causes mitosis defects, but does not directly target tubulin or actin. The *ent*-Kauranoids might target common protein(s) involved in Smo ciliary transport, ciliogenesis and mitosis. Similar to the *ent*-Kauranoids, ciliobrevin (HPI–4) has been shown to inhibit Hh signaling while simultaneously affecting cilia length, Gli2 localization and spindle formation [25, 38]. Subsequent work demonstrated that it targets the cytoplasmic motor protein dynein, which is involved in ciliary trafficking, mitotic spindle formation and organelle transport [24].

In addition to dynein, there are other proteins that are reported to participate in both cilia formation and mitosis, such as the IFT motor protein Kif3a [39, 40], the IFT complex component IFT88 [40, 41], and the oncogenic Aurora A kinase [42, 43]. Specifically, the Ran importin system was recently found to regulate ciliogenesis [44], which forms a diffusion barrier at the base of cilia to selectively control the entry of proteins into cilia [45, 46]. The Ran importin system is also critical for the accurate progression of mitosis, playing roles in spindle microtubule assembly, kinetochore-microtubule attachment and chromosome congression [47]. Interestingly, *ent*-15-oxokaurenoic acid, a chemical with similar backbone to the *ent*-Kauranoids, has been reported to cause mitotic arrest and defective chromosome congression by targeting RanBP2 [48], a Ran binding protein from the Ran importin system. Additionally, RanBP9 (RanBPM) and RanBP10 have been identified as Smo-binding proteins, with RanBP9 being involved in the cilia transportation of Smo [49]. Thus, the Ran binding proteins are attractive candidate targets for *ent*-Kauranoids, a possibility that awaits further investigation.

Maintenance of normal ciliary length is critical for the diverse functions of cilia including the Hh signal transduction, which has been reported to be affected by many proteins and compounds [25, 33, 50, 51]. The *ent*-Kaurane diterpenoids we identified might provide new tools for deciphering the mechanisms of ciliogenesis and cilia-dependent regulation of the Hh pathway.

## Supporting Information

S1 Fig IC_50_s of all the HPAs in SAG activated conditions. The IC_50_ values and curves were determined with GraphPad Prism 6 based on the sigmoid dose-response analysis with a variable slope. The data are presented as the average of triplicate samples ± SD.
(TIF)

S2 Fig The HPAs do not inhibit Wnt signaling. HEK 293W cells were treated with HPAs at 10 μM for 1 day before being processed for luciferase activity analysis. EGCG is a known Wnt inhibitor. The data are presented as the average of triplicate samples ± SD. Asterisks indicate *p* < 0.05 for individual compounds vs. DMSO.
(TIF)

S3 Fig NF-κB inhibitors do not suppress Hh signaling. The effects of the NF-κB inhibitors AZ-TAK1 and 5Z-7-Oxoeaneol on SAG activated Hh signaling were tested in Shh light II cells., The data are presented as the average of triplicate samples ± SD. Asterisks indicate *p* < 0.05 for individual compounds vs. SAG.
(TIF)

S4 Fig Binuclei and micronuclei were observed in kamebakaurin treated cells. Binuclei or micronuclei were frequently observed in NIH 3T3 cells treated with kamebakaurin (3 μM) for 30 hr. The asterisks indicate the micronuclei. Scale bar: 5 μm.
(TIF)

S5 Fig Defective spindle assembly and chromosome alignment in cells treated with kamebakaurin at 25 μM. Spindles were poorly organized In NIH 3T3 cells treated with 25 μM kamebakaurin, although less severely than in cells treated with 50 μM kamebakaurin, where the chromosomes were misaligned. The centrosomal protein pericentrin showed a diffused distribution (asterisks). Scale bar: 5 μm.
(TIF)

## References

[1] Briscoe J, Therond PP. The mechanisms of Hedgehog signalling and its roles in development and disease. Nat Rev Mol Cell Biol 2013; 14: 416–429. 10.1038/nrm3598 . 23719536

[2] Amakye D, Jagani Z, Dorsch M. Unraveling the therapeutic potential of the Hedgehog pathway in cancer. Nat Med 2013; 19: 1410–1422. 10.1038/nm.3389 . 24202394

[3] Chang AL, Oro AE. Initial assessment of tumor regrowth after vismodegib in advanced Basal cell carcinoma. Arch Dermatol 2012; 148: 1324–1325. 10.1001/archdermatol.2012.2354 . 22910979 PMC3777384

[4] Rudin CM, Hann CL, Laterra J, Yauch RL, Callahan CA, Fu L, et al Treatment of Medulloblastoma with Hedgehog Pathway Inhibitor GDC–0449. N Engl J Med 2009; 361: 1173–1178. 10.1056/NEJMoa0902903 . 19726761 PMC5317279

[5] Sharpe HJ, Pau G, Dijkgraaf GJ, Basset-Seguin N, Modrusan Z, Januario T, et al Genomic analysis of smoothened inhibitor resistance in basal cell carcinoma. Cancer Cell 2015; 27: 327–341. 10.1016/j.ccell.2015.02.001 . 25759019 PMC5675004

[6] Yauch RL, Dijkgraaf GJ, Alicke B, Januario T, Ahn CP, Holcomb T, et al Smoothened mutation confers resistance to a Hedgehog pathway inhibitor in medulloblastoma. Science 2009; 326: 572–574. 10.1126/science.1179386 . 19726788 PMC5310713

[7] Goetz SC, Anderson KV. The primary cilium: a signalling centre during vertebrate development. Nat Rev Genet 2010; 11: 331–344. 10.1038/nrg2774 . 20395968 PMC3121168

[8] Nozawa YI, Lin C, Chuang PT. Hedgehog signaling from the primary cilium to the nucleus: an emerging picture of ciliary localization, trafficking and transduction. Curr Opin Genet Dev 2013; 23: 429–437. 10.1016/j.gde.2013.04.008 . 23725801 PMC3913210

[9] Han YG, Kim HJ, Dlugosz AA, Ellison DW, Gilbertson RJ, Alvarez-Buylla A. Dual and opposing roles of primary cilia in medulloblastoma development. Nat Med 2009; 15: 1062–1065. 10.1038/nm.2020 . 19701203 PMC2771737

[10] Wong SY, Seol AD, So PL, Ermilov AN, Bichakjian CK, Epstein EH Jr., et al Primary cilia can both mediate and suppress Hedgehog pathway-dependent tumorigenesis. Nat Med 2009; 15: 1055–1061. 10.1038/nm.2011 . 19701205 PMC2895420

[11] Sharpe HJ, Wang W, Hannoush RN, de Sauvage FJ. Regulation of the oncoprotein Smoothened by small molecules. Nat Chem Biol 2015; 11: 246–255. 10.1038/nchembio.1776 . 25785427

[12] Cragg GM, Newman DJ. Natural products: a continuing source of novel drug leads. Biochim Biophys Acta 2013; 1830: 3670–3695. 10.1016/j.bbagen.2013.02.008 . 23428572 PMC3672862

[13] Chen JK, Taipale J, Cooper MK, Beachy PA. Inhibition of Hedgehog signaling by direct binding of cyclopamine to Smoothened. Genes Dev 2002; 16: 2743–2748. 10.1101/gad.1025302 . 12414725 PMC187469

[14] Taipale J, Chen JK, Cooper MK, Wang B, Mann RK, Milenkovic L, et al Effects of oncogenic mutations in Smoothened and Patched can be reversed by cyclopamine. Nature 2000; 406: 1005–1009. 10.1038/35023008 . 10984056

[15] Li XY, Wang YY, Yuan CM, Hao XJ, Li Y. A reporter gene system for screening inhibitors of Wnt signaling pathway. Nat Prod Bioprospect 2013; 3: 24–28. 10.1007/s13659-012-0094-0

[16] Svärd J, Heby-Henricson K, Persson-Lek M, Rozell B, Lauth M, Bergström A, et al Genetic elimination of Suppressor of fused reveals an essential repressor function in the mammalian Hedgehog signaling pathway. Dev Cell 2006; 10: 187–197. 10.1016/j.devcel.2005.12.013 . 16459298

[17] Yue S, Chen Y, Cheng SY. Hedgehog signaling promotes the degradation of tumor suppressor Sufu through the ubiquitin-proteasome pathway. Oncogene 2009; 28: 492–499. 10.1038/onc.2008.403 . 18997815

[18] Yoshio T, Teruyoshi I, Yoshihisa T, and Tetsuro F. Structural elucidation of new diterpenoids isolated from Rabdosia umbrosa var. Ieucantha f. kameba. J Chem Soc Perkin Trans 1987; 1: 2403–2409.

[19] Li X, Xiao WL, Pu JX, Ban LL, Shen YH, Weng ZY, et al Cytotoxic ent-kaurene diterpenoids from Isodon phyllostachys. Phytochemistry 2006; 67: 1336–1340. 10.1016/j.phytochem.2006.05.002 . 16777159

[20] Chen YP, and Sun HD. Study on the Rabdosia calcicolus var. subcalvus. Acta Botanica Yunnanica 1990; 12: 211–217.

[21] Yang JH, Du X, He F, Zhang HB, Li XN, Su J, et al Bioactive abietane and ent-kaurane diterpenoids from Isodon tenuifolius. J Nat Prod 2013; 76: 256–264. 10.1021/np300772e . 23327668

[22] Jiang B, Yang H, Li ML, Hou AJ, Han QB, Wang SJ, et al Diterpenoids from Isodon adenantha. J Nat Prod 2002; 65: 1111–1116. . 12193013 10.1021/np020084k

[23] Yang JH, Pu JX, Wen J, Li XN, He F, Xue YB, et al Cytotoxic triterpene dilactones from the stems of Kadsura ananosma. J Nat Prod 2010; 73: 12–16. 10.1021/np900506g . 20025236

[24] Pu JX, Li RT, Xiao WL, Gong NB, Huang SX, Lu Y, et al Longipedlactones A-I, nine novel triterpene dilactones possessing a unique skeleton from Kadsura longipedunculata. Tetrahedron 2006; 62: 6073–6081. 10.1016/j.tet.2006.03.108

[25] Firestone AJ, Weinger JS, Maldonado M, Barlan K, Langston LD, O'Donnell M, et al Small-molecule inhibitors of the AAA+ ATPase motor cytoplasmic dynein. Nature 2012; 484: 125–129. 10.1038/nature10936 . 22425997 PMC3321072

[26] Chen JK, Taipale J, Young KE, Maiti T, Beachy PA. Small molecule modulation of Smoothened activity. Proc Natl Acad Sci USA 2002; 99: 14071–14076. 10.1073/pnas.182542899 . 12391318 PMC137838

[27] Wang L, Li D, Wang C, Zhang Y, Xu J. Recent progress in the development of natural ent-kaurane diterpenoids with anti-tumor activity. Mini Rev Med Chem 2011; 11: 910–919. . 21781025 10.2174/138955711796575416

[28] Nachtergaele S, Whalen DM, Mydock LK, Zhao Z, Malinauskas T, Krishnan K, et al Structure and function of the Smoothened extracellular domain in vertebrate Hedgehog signaling. eLife 2013; 2: e01340 10.7554/eLife.01340 24171105 PMC3809587

[29] Cooper AF, Yu KP, Brueckner M, Brailey LL, Johnson L, McGrath JM, et al Cardiac and CNS defects in a mouse with targeted disruption of suppressor of fused. Development 2005; 132: 4407–4417. 10.1242/dev.02021 . 16155214

[30] Lee JH, Koo TH, Hwang BY, Lee JJ. Kaurane diterpene, kamebakaurin, inhibits NF-kappa B by directly targeting the DNA-binding activity of p50 and blocks the expression of antiapoptotic NF-kappa B target genes. J Biol Chem 2002; 277: 18411–18420. 10.1074/jbc.M201368200 . 11877450

[31] Buglio D, Palakurthi S, Byth K, Vega F, Toader D, Saeh J, et al Essential role of TAK1 in regulating mantle cell lymphoma survival. Blood 2012; 120: 347–355. 10.1182/blood-2011-07-369397 . 22649101 PMC3460632

[32] Wu J, Powell F, Larsen NA, Lai Z, Byth KF, Read J, et al Mechanism and in vitro pharmacology of TAK1 inhibition by (5Z)-7-Oxozeaenol. ACS Chem Biol 2013; 8: 643–650. 10.1021/cb3005897 . 23272696

[33] Ishikawa H, Marshall WF. Ciliogenesis: building the cell's antenna. Nat Rev Mol Cell Biol 2011; 12: 222–234. 10.1038/nrm3085 . 21427764

[34] He M, Subramanian R, Bangs F, Omelchenko T, Liem KF Jr., Kapoor TM, et al The kinesin–4 protein Kif7 regulates mammalian Hedgehog signalling by organizing the cilium tip compartment. Nat Cell Biol 2014; 16: 663–672. 10.1038/ncb2988 . 24952464 PMC4085576

[35] Liem KF Jr., He M, Ocbina PJ, Anderson KV. Mouse Kif7/Costal2 is a cilia-associated protein that regulates Sonic hedgehog signaling. Proc Natl Acad Sci USA 2009; 106: 13377–13382. 10.1073/pnas.0906944106 . 19666503 PMC2726420

[36] Endoh-Yamagami S, Evangelista M, Wilson D, Wen X, Theunissen JW, Phamluong K, et al The mammalian Cos2 homolog Kif7 plays an essential role in modulating Hh signal transduction during development. Curr Biol 2009; 19: 1320–1326. 10.1016/j.cub.2009.06.046 . 19592253

[37] Cheung HO, Zhang X, Ribeiro A, Mo R, Makino S, Puviindran V, et al The kinesin protein Kif7 is a critical regulator of Gli transcription factors in mammalian hedgehog signaling. Sci Signal 2009; 2: ra29 10.1126/scisignal.2000405 . 19549984

[38] Hyman JM, Firestone AJ, Heine VM, Zhao Y, Ocasio CA, Han K, et al Small-molecule inhibitors reveal multiple strategies for Hedgehog pathway blockade. Proc Natl Acad Sci U S A 2009; 106: 14132–14137. 10.1073/pnas.0907134106 . 19666565 PMC2721821

[39] Haraguchi K, Hayashi T, Jimbo T, Yamamoto T, Akiyama T. Role of the kinesin–2 family protein, KIF3, during mitosis. J Biol Chem 2006; 281: 4094–4099. 10.1074/jbc.M507028200 . 16298999

[40] Huangfu D, Liu A, Rakeman AS, Murcia NS, Niswander L, Anderson KV. Hedgehog signalling in the mouse requires intraflagellar transport proteins. Nature 2003; 426: 83–87. 10.1038/nature02061 . 14603322

[41] Delaval B, Bright A, Lawson ND, Doxsey S. The cilia protein IFT88 is required for spindle orientation in mitosis. Nat Cell Biol 2011; 13: 461–468. 10.1038/ncb2202 . 21441926 PMC3073523

[42] Pugacheva EN, Jablonski SA, Hartman TR, Henske EP, Golemis EA. HEF1-dependent Aurora A activation induces disassembly of the primary cilium. Cell 2007; 129: 1351–1363. 10.1016/j.cell.2007.04.035 . 17604723 PMC2504417

[43] Fu JY, Bian ML, Jiang Q, Zhang CM. Roles of Aurora kinases in mitosis and tumorigenesis. Mol Cancer Res 2007; 5: 1–10. 10.1158/1541-7786.mcr-06-0208 . 17259342

[44] Fan S, Whiteman EL, Hurd TW, McIntyre JC, Dishinger JF, Liu CJ, et al Induction of Ran GTP drives ciliogenesis. Mol Biol Cell 2011; 22: 4539–4548. 10.1091/mbc.E11-03-0267 . 21998203 PMC3226473

[45] Dishinger JF, Kee HL, Jenkins PM, Fan S, Hurd TW, Hammond JW, et al Ciliary entry of the kinesin–2 motor KIF17 is regulated by importin-beta2 and RanGTP. Nat Cell Biol 2010; 12: 703–710. 10.1038/ncb2073 . 20526328 PMC2896429

[46] Kee HL, Dishinger JF, Blasius TL, Liu CJ, Margolis B, Verhey KJ. A size-exclusion permeability barrier and nucleoporins characterize a ciliary pore complex that regulates transport into cilia. Nat Cell Biol 2012; 14: 431–437. 10.1038/ncb2450 . 22388888 PMC3319646

[47] Clarke PR, Zhang C. Spatial and temporal coordination of mitosis by Ran GTPase. Nat Rev Mol Cell Biol 2008; 9: 464–477. 10.1038/nrm2410 . 18478030

[48] Rundle NT, Nelson J, Flory MR, Joseph J, Th'ng J, Aebersold R, et al An ent-kaurene that inhibits mitotic chromosome movement and binds the kinetochore protein ran-binding protein 2. ACS Chem Biol 2006; 1: 443–450. 10.1021/cb600196w . 17168522

[49] Xie JW, Sheng T, Zhang XL. Hedgehog signaling pathway proteins and uses thereof. U.S. patent 2010; 20100111955 A1.

[50] Besschetnova TY, Kolpakova-Hart E, Guan Y, Zhou J, Olsen BR, Shah JV. Identification of signaling pathways regulating primary cilium length and flow-mediated adaptation. Curr Biol 2010; 20: 182–187. 10.1016/j.cub.2009.11.072 . 20096584 PMC2990526

[51] Kim J, Lee JE, Heynen-Genel S, Suyama E, Ono K, Lee K, et al Functional genomic screen for modulators of ciliogenesis and cilium length. Nature 2010; 464: 1048–1051. 10.1038/nature08895 . 20393563 PMC2929961

