# Supplementary figures and images for: A Group of ent-Kaurane Diterpenoids Inhibit Hedgehog Signaling and Induce Cilia Elongation
Source: PLoS One. 2015 Oct 6;10(10):e0139830. doi: 10.1371/journal.pone.0139830 (PMC4595341; doi:10.1371/journal.pone.0139830)

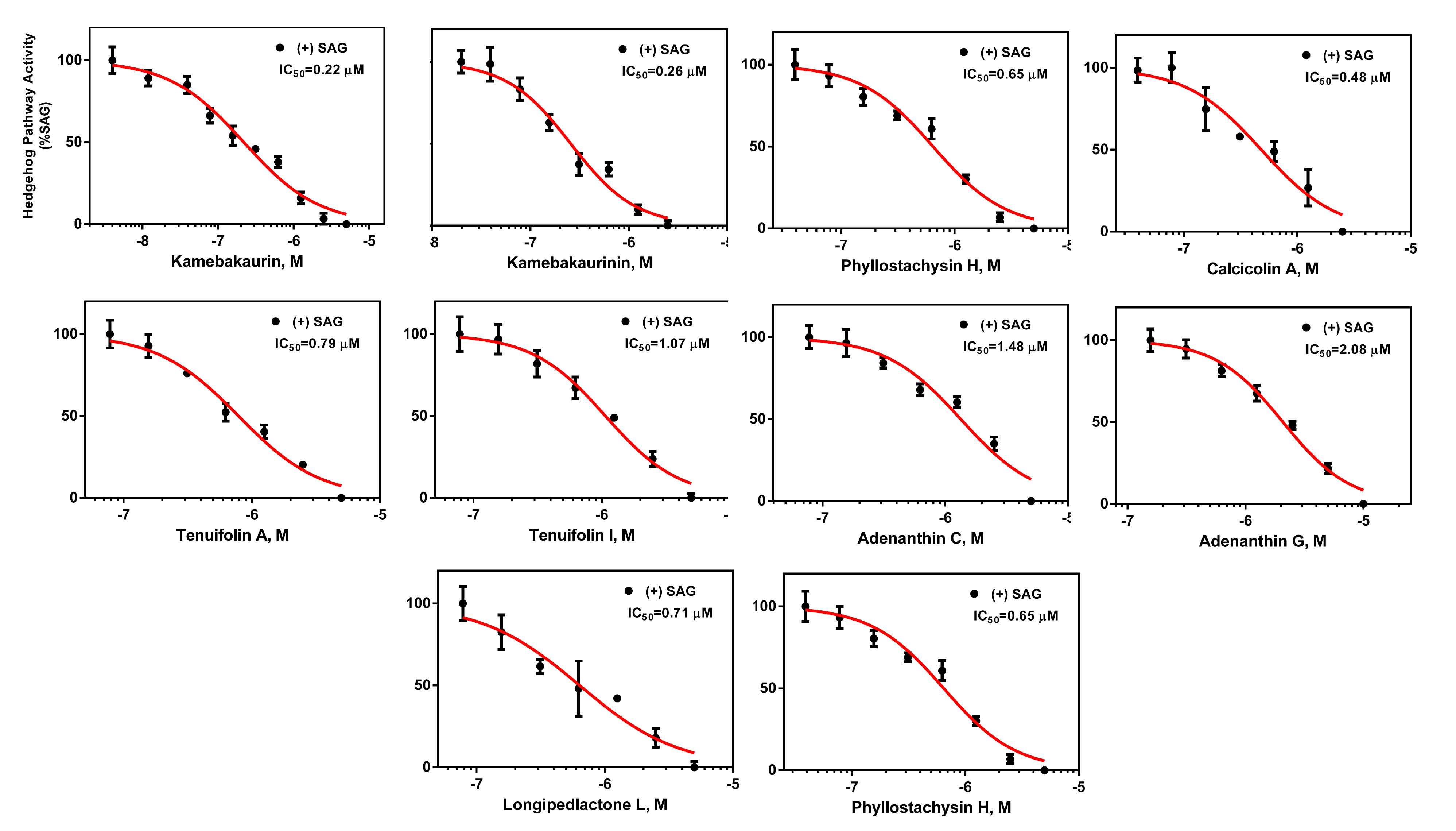

Supplement: S1 Fig — The IC50 values and curves were determined with GraphPad Prism 6 based on the sigmoid dose-response analysis with a variable slope. The data are presented as the average of triplicate samples ± SD. (TIF) [file pone.0139830.s001.tif]

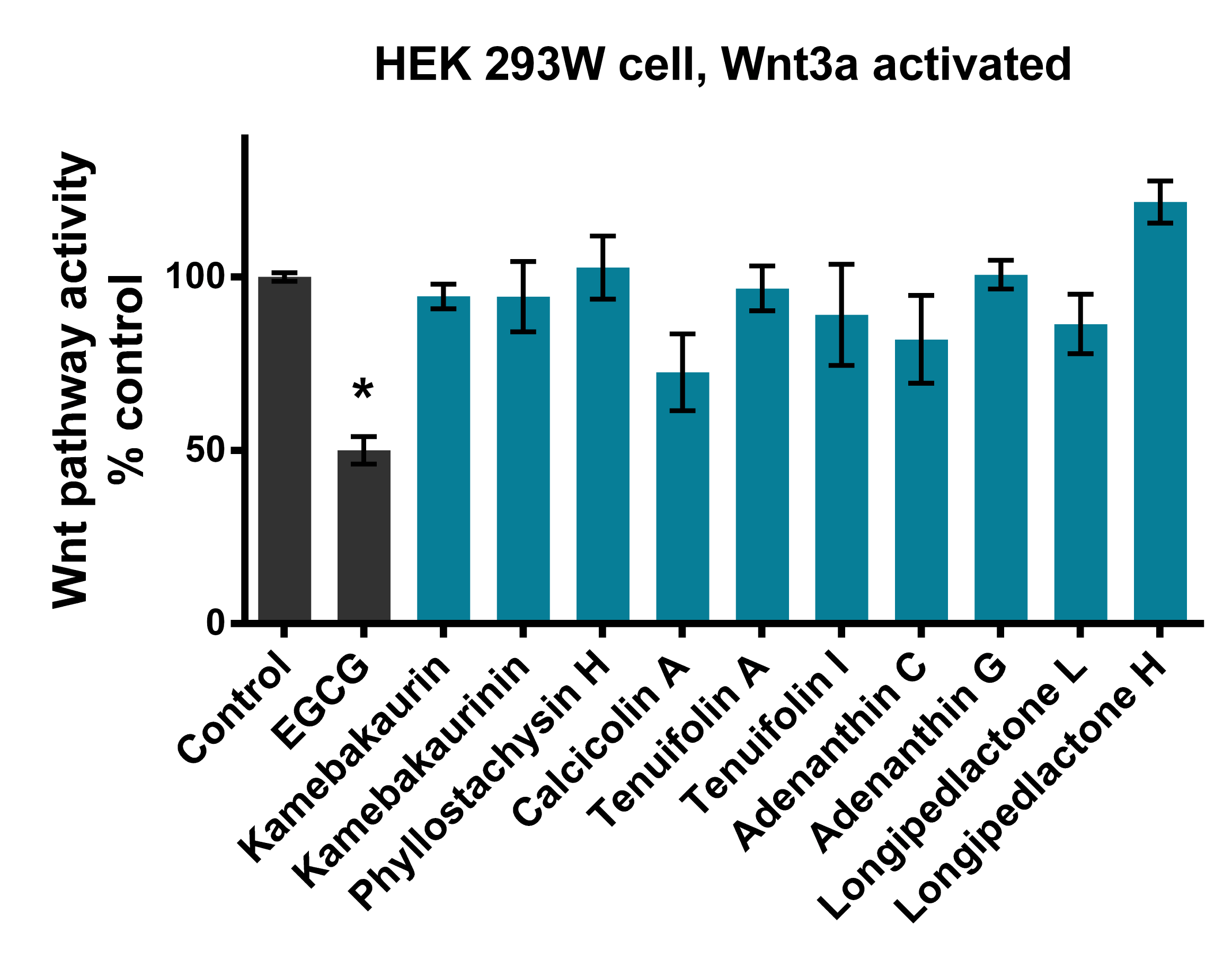

Supplement: S2 Fig — HEK 293W cells were treated with HPAs at 10 μM for 1 day before being processed for luciferase activity analysis. EGCG is a known Wnt inhibitor. The data are presented as the average of triplicate samples ± SD. Asterisks indicate p < 0.05 for individual compounds vs. DMSO. (TIF) [file pone.0139830.s002.tif]

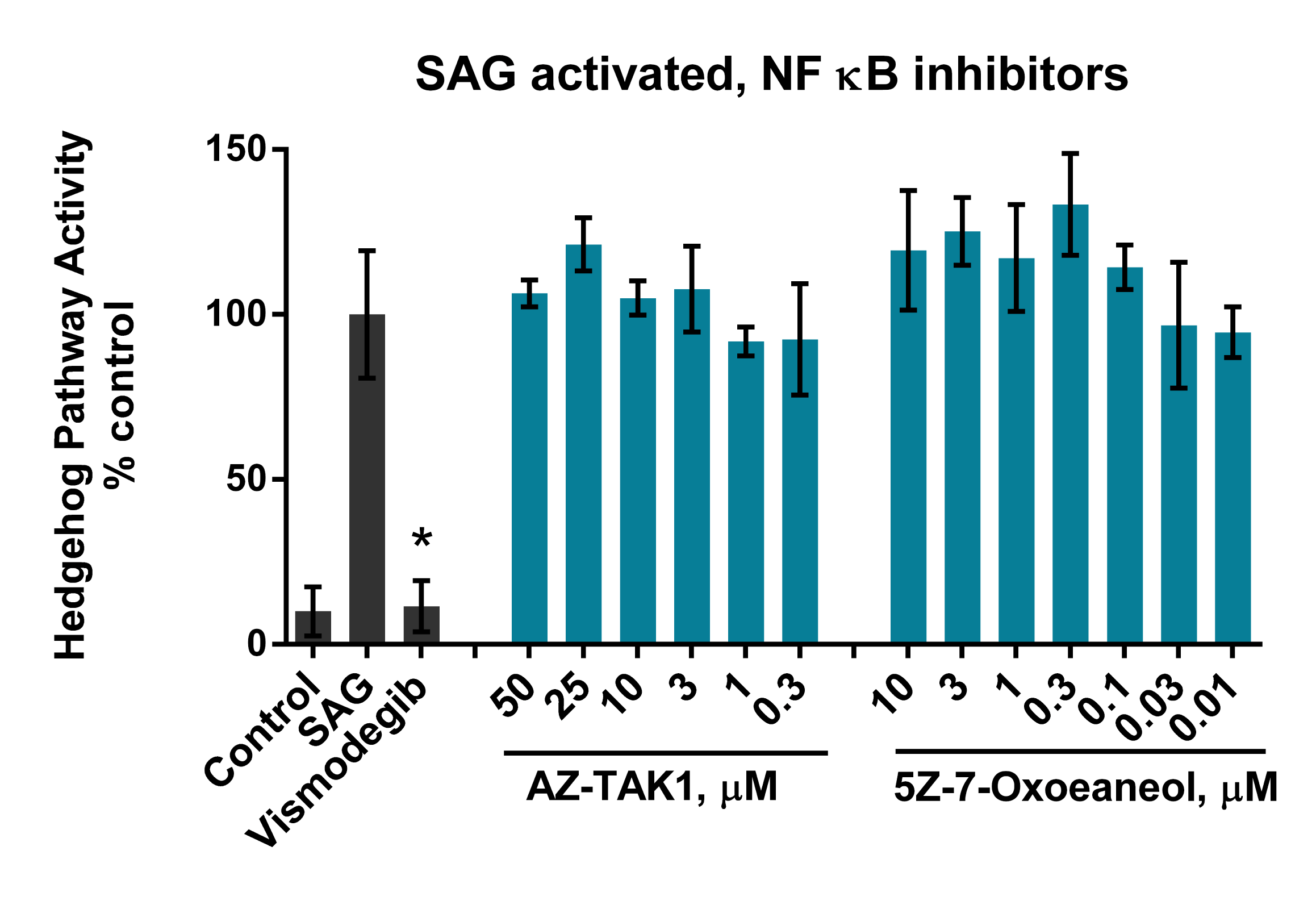

Supplement: S3 Fig — The effects of the NF-κB inhibitors AZ-TAK1 and 5Z-7-Oxoeaneol on SAG activated Hh signaling were tested in Shh light II cells., The data are presented as the average of triplicate samples ± SD. Asterisks indicate p < 0.05 for individual compounds vs. SAG. (TIF) [file pone.0139830.s003.tif]

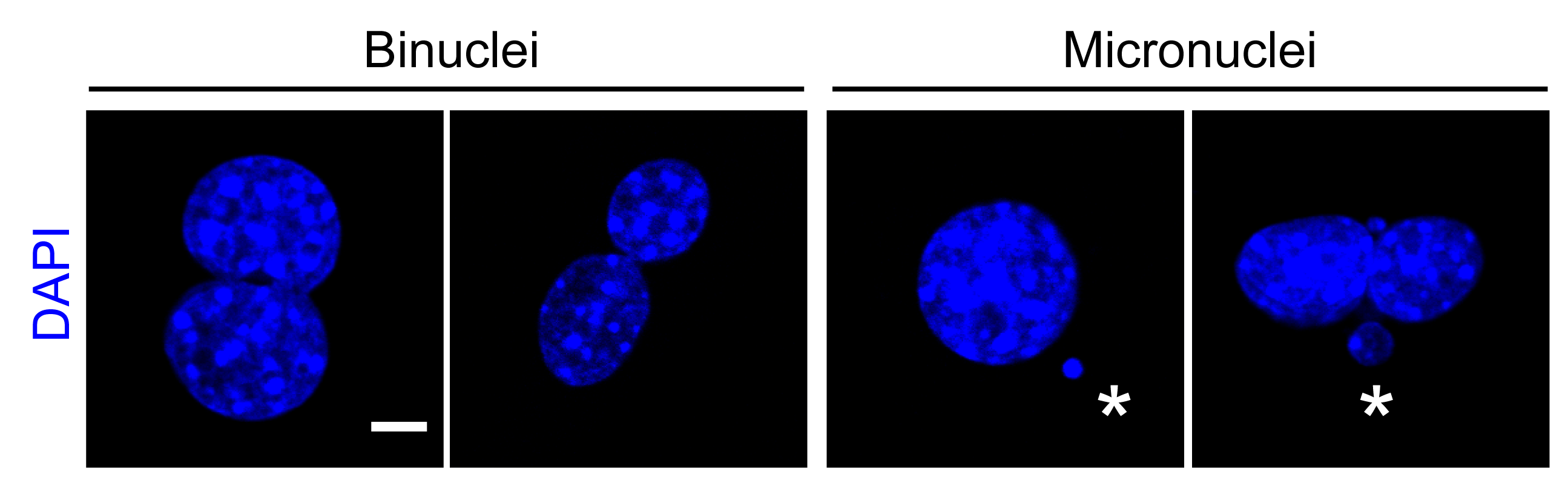

Supplement: S4 Fig — Binuclei or micronuclei were frequently observed in NIH 3T3 cells treated with kamebakaurin (3 μM) for 30 hr. The asterisks indicate the micronuclei. Scale bar: 5 μm. (TIF) [file pone.0139830.s004.tif]

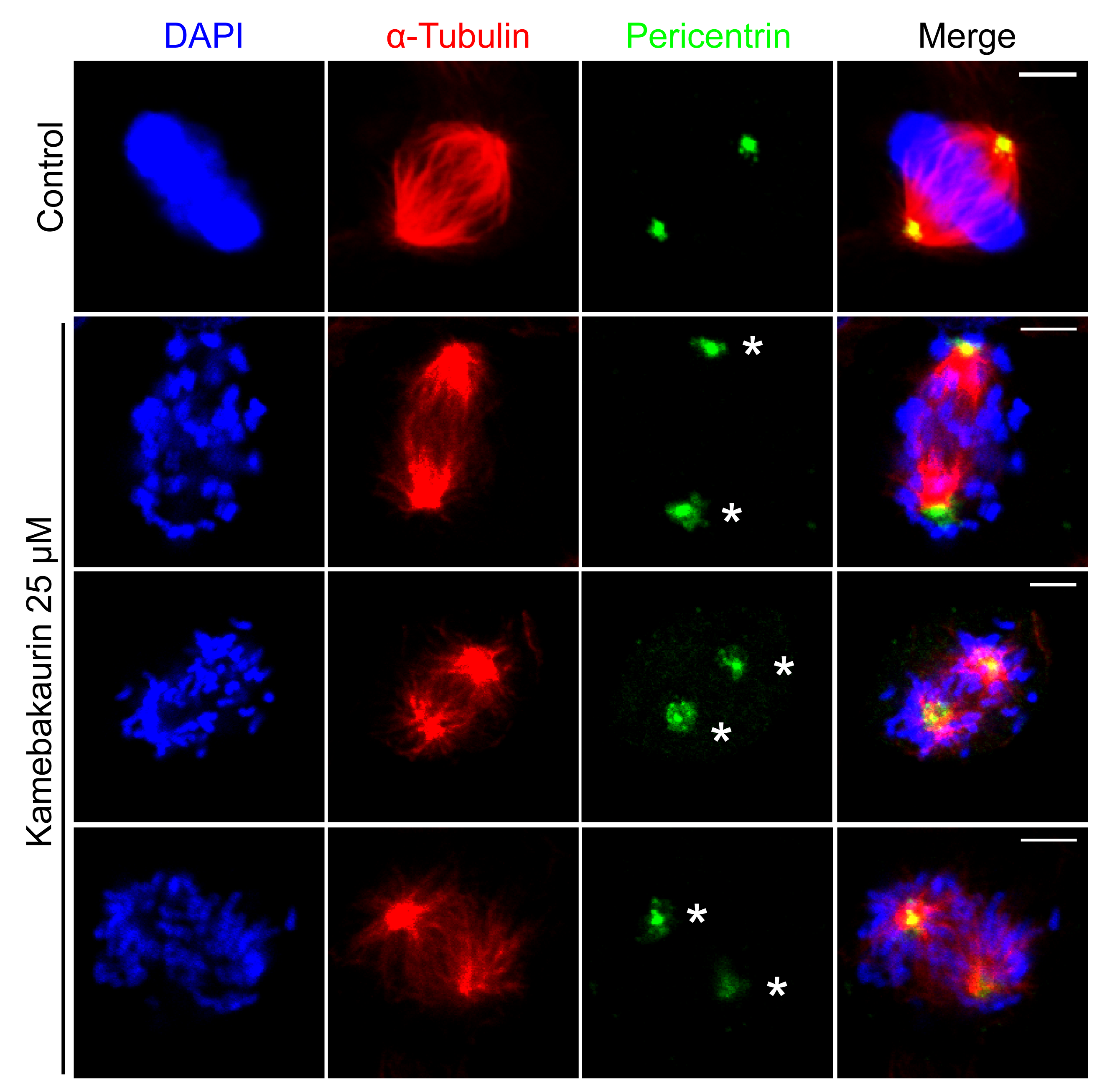

Supplement: S5 Fig — Spindles were poorly organized In NIH 3T3 cells treated with 25 μM kamebakaurin, although less severely than in cells treated with 50 μM kamebakaurin, where the chromosomes were misaligned. The centrosomal protein pericentrin showed a diffused distribution (asterisks). Scale bar: 5 μm. (TIF) [file pone.0139830.s005.tif]
